# Single-neuron mechanisms of neural adaptation in the human temporal lobe

**DOI:** 10.1038/s41467-023-38190-5

**Published:** 2023-04-29

**Authors:** Thomas P. Reber, Sina Mackay, Marcel Bausch, Marcel S. Kehl, Valeri Borger, Rainer Surges, Florian Mormann

**Affiliations:** 1 Faculty of Psychology, UniDistance Suisse, Brig, Switzerland; 2 Department of Epileptology, University of Bonn Medical Centre, Bonn, Germany; 3 Department of Neurosurgery, University of Bonn Medical Centre, Bonn, Germany

**Keywords:** Habituation, Human behaviour, Hippocampus

## Abstract

A central function of the human brain is to adapt to new situations based on past experience. Adaptation is reflected behaviorally by shorter reaction times to repeating or similar stimuli, and neurophysiologically by reduced neural activity in bulk-tissue measurements with fMRI or EEG. Several potential single-neuron mechanisms have been hypothesized to cause this reduction of activity at the macroscopic level. We here explore these mechanisms using an adaptation paradigm with visual stimuli bearing abstract semantic similarity. We recorded intracranial EEG (iEEG) simultaneously with spiking activity of single neurons in the medial temporal lobes of 25 neurosurgical patients. Recording from 4917 single neurons, we demonstrate that reduced event-related potentials in the macroscopic iEEG signal are associated with a sharpening of single-neuron tuning curves in the amygdala, but with an overall reduction of single-neuron activity in the hippocampus, entorhinal cortex, and parahippocampal cortex, consistent with fatiguing in these areas.

## Abstract (web summary)

Behavioural adaptation from semantic priming is accompanied by reduced neural activity in bulk-tissue measurements, but the underlying single neuron mechanisms remain unclear. Here, the authors leverage simultaneous intracranial EEG and single neuron spiking recordings in the human medial temporal lobe to unveil differential sharpening and fatiguing mechanisms across different temporal lobe areas.

## Introduction

A vital capacity for an organism to survive and reproduce is to adapt behavior based on prior experience. One of the most fundamental forms of memory that facilitates these behavioral adaptations manifests in faster and more efficient processing of repeating external stimuli^1^ – a behavior that can be observed throughout the animal kingdom, and even in organism lacking a central nervous system^2^. In more complex organisms, behavioral adaptation is achieved not only on repeated exposure to identical stimuli, but also when a feature of novel experience merely bears resemblance to past experience at an increasingly abstract level. Arguably unique to human is adaptation based on similarity at the level of abstract semantics. While behavioral adaptation to abstract semantics is at the heart of human cognition, little is known about the neuronal mechanisms achieving this feat.

Behavioral adaptation is also referred to as priming and has been considered a fundamental, implicit form of memory. Behavioral adaptation is often accompanied by a reduction in neural activity for repeated stimuli, i.e., repetition priming^3^, or for perceptually or conceptually similar stimuli, i.e., relatedness priming. Neural activity reduction during behavioral facilitation has been referred to as “neural priming”, “neural adaptation” or “repetition suppression”. Neural adaptation (NA) has more recently been framed as a correlate of predictive signals that are ubiquitous in the brain^4^. NA can be observed with coarse measures of neural activity such as fMRI, MEG, EEG, and intracranial EEG^5–7^. The mechanisms at the micro-level of single neurons that give rise to NA at the meso- and macro-level (iEEG, and fMRI/EEG), however, remain elusive^3,8,9^.

Psychological and computational models for semantic relatedness priming have been formulated as early as the 1960’s^10,11^. Likely the most influential models are spreading activation models that assume semantic knowledge to be stored in a network with nodes representing individual concepts or semantic features thereof. Nearby concepts and features in semantic space are assumed to be connected through fewer nodes in the network. In these models, facilitation of behavioral responses in conceptual relatedness priming tasks is the consequence of residual activity spreading to neighboring nodes in the network, essentially lowering the threshold of activation in neighboring nodes. The spreading activation model has been influential in linguistic and behavioral cognitive research^12^. With a few exceptions pertaining to neurotransmitter levels modulating the amount of spreading activation^13,14^, the precise biological implementation and plausibility of the spreading activation model have not been sufficiently addressed. Furthermore, the spreading activation model does not explain how NA on meso- and macroscopic measures of brain activity emerges from activity patterns in populations of single neurons.

In the context of repetition rather than relatedness priming, several single-unit mechanisms that may give rise to NA at the macro-level have been hypothesized^3,8,9,15,16^. One of them is referred to as sharpening. At the level of an individual neuron, sharpening describes a situation in which a neuron keeps firing as strongly to its optimal stimulus on repeated versus initial presentations while it reduces firing disproportionally in response to non-optimal stimuli^17,18^. Apart from this sharpening of tuning curves of individual units, sharpening at the population level describes the situation that in repeated presentations for the same stimulus, fewer neurons fire than during the initial presentation^19^. Fatiguing is observed when neurons reduce their firing rate proportionally to all response-eliciting stimuli without affecting the shape of the tuning curve^20–22^. Both, sharpening and fatiguing reduce activity to non-optimal stimuli and by this increase stimulus selectivity of the population response. Increased selectivity is then thought to be responsible for faster propagation of signals to downstream regions and by this essentially enable behavioral facilitation. A shortening of periods of neuronal activity in response to repeated versus initial presentations is referred to as facilitation^23^. A consequence of facilitation in down-stream regions of the processing path is a reduction in the latency with which neurons respond to a stimulus. The neuronal facilitation model nicely explains observations of shortened reaction times at the behavioral level. Despite its appeal, evidence in favor for the facilitation model on the level of single-neuron data is lacking. More recently, however, two intrancranial EEG studies have reported evidence of earlier peak latencies in evoked activity during priming^24,25^. Investigations of potential single-unit mechanisms of NA, however, so far have been mostly confined to studies of visual perception and object recognition in primary sensory areas and area IT in non-human primates^17,18,21,22^. As the sharpening, fatiguing and facilitation models have been formulated in the context of repetition priming, it remains open whether and under what circumstances fatiguing, facilitation or sharpening can be observed as underlying mechanism for NA in conceptual relatedness priming humans and in areas associated with higher cognitive functions.

The human MTL is a prime candidate to assess these questions as it is a brain region that has been implicated in several of higher forms of cognition in humans and from which single neuron and intracranial EEG data can be recorded simultaneously in the rare occasions given by invasive epilepsy monitoring^26^. Single neuron data from the human MTL have been instrumental to further our understanding of higher forms of cognition such as episodic and semantic memory^27–30^, working memory^31,32^, spatial navigation^33,34^, as well as emotion recognition and appraisal^35–37^. NA in the human MTL has not only been demonstrated on identical repetitions of visual stimuli^8,38,39^ but also on target stimuli following semantically similar versus dissimilar prime stimuli^40,41^. NA in the MTL following semantic priming dovetails with the notion of a primarily semantic neuronal code of single units in humans^28,35,42–45^.

The aim of the current work is to investigate the single-unit mechanisms of NA in the human MTL following behavioral adaptation to highly abstract semantic information. We recorded simultaneous iEEG and single-unit activity from the MTLs of patients undergoing chronic epilepsy monitoring while they performed a semantic priming experiment.

## Results

### Adaptation to semantics manifests in behavioral measures

**Fig. 1:**
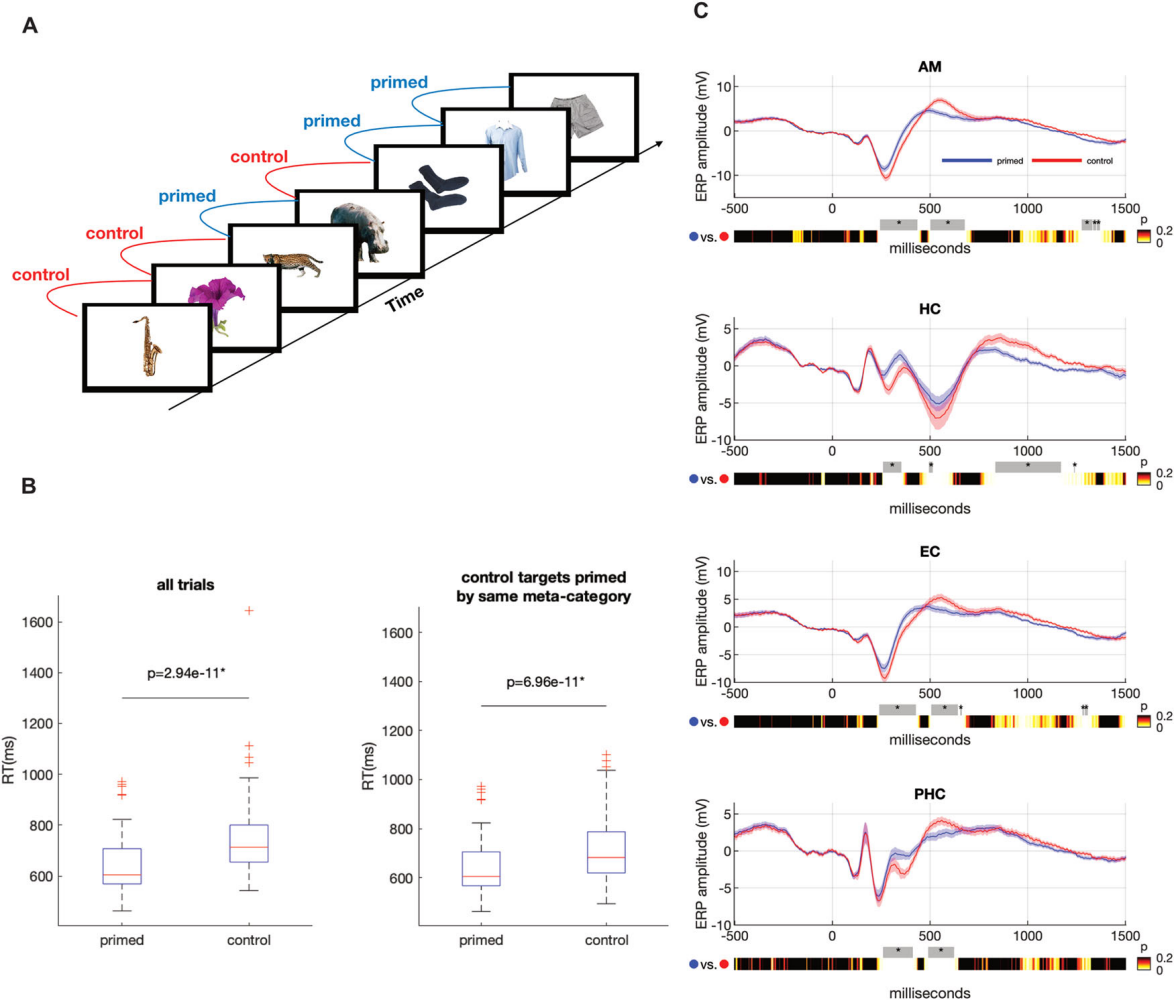
Design, ERPs, and behavioral data. **A** Participants performed a manmade vs. natural decision on images presented in a sequence that was arranged such that half of the images were preceded by the same/different category (primed/control condition). **B** Behavioral priming is observed in significant differences in mean reaction time differences (RT) between primed and control targets. Sample size *n* = 59 sessions. *P*-values are derived from an uncorrected Wilcoxon Signed Ranks test. The left plot includes all trials (*p* = 2.94 × 10^−11^, *Z* = 4), whereas the right plot only includes control targets that were preceded by prime stimuli affording the same behavioral response (*p* = 6.96 × 10^−11^, *Z* = 21; manmade/natural meta-category), excluding mere response priming. Center line in boxplots denote the median. Box limits denote the upper and lower quartiles. Whiskers denote the median ± 1.5x interquartile range and data points exceeding the whiskers are shown as red crosses. Source data are provided as Source Data file (SourceData.xlsx). **C** Grand-average ERPs of the most medial contacts of intracranial electrodes were computed for the amygdala (AM), entorhinal cortex (EC), hippocampus (HC), and parahippocampal cortex (PHC). Grand average ERPs as depicted were calculated from individual ERPs derived per experimental session, averaging over electrodes in anatomical regions across the two hemispheres. As implantation schemes varied between participants, sample sizes for calculating grand average ERPs vary between anatomical regions (*n* = 59 sessions for AM, *n* = 57 sessions in HC, *n* = 49 sessions in EC, *n* = 52 sessions in PHC). Colored bars depict the uncorrected *p*-values of two-tailed *t*-tests performed at each sample point. Shaded colored areas denote the standard error of the mean. Gray bars with an asterisk on top denote significant differences according to a two-sided cluster-based permutation test^46^. More detail on significant clusters is given in the following. AM primed > control from 244 ms to 435 ms, clustersize = 49, sum of t-value *s* = 324.95, *p* < 0.001; primed < control from 502 ms to 678 ms, clustersize = 45, sum of t-value *s* = −271.5, *p* < 0.001; from 1276 ms to 1331 ms, clustersize = 14, sum of t-value *s* = −63.208, *p* < 0.001; from 1335 ms to 1351 ms, clustersize = 4, sum of t-value *s* = −16.601, *p* < 0.001; from 1355 ms to 1370 ms, clustersize = 4, sum of t-value *s* = −14.708, *p* = 0.002; HC primed > control from 259 ms to 353 ms, clustersize = 24, sum of t-value *s* = 150.24, *p* < 0.001; from 494 ms to 514 ms, clustersize = 5, sum of t-value *s* = 17.576, *p* < 0.001; primed < control from 834 ms to 1171 ms, clustersize = 86, sum of t-value *s* = −382.66, *p* < 0.001; from 1237 ms to 1241 ms, clustersize = 1, sum of t-value *s* = −3.6714, *p* = 0.002; EC primed > control from 240 ms to 428 ms, clustersize = 48, sum of t-value *s* = 261.15, *p* < 0.001; primed <control from 506 ms to 643 ms, clustersize = 35, sum of t-value *s* = −181.6, *p* < 0.001; from 654 ms to 662 ms, clustersize = 2, sum of t-value *s* = −7.599, *p* < 0.001; from 1280 ms to 1288 ms, clustersize = 2, sum of t-value *s* = −7.8629, *p* < 0.001; from 1292 ms to 1308 ms, clustersize = 4, sum of t-value *s* = −16.11, *p* < 0.001; PHC primed > control from 259 ms to 412 ms, clustersize = 39, sum of t-value *s* = 210.88, *p* < 0.001; primed <control from 490 ms to 623 ms, clustersize = 34, sum of t-value *s* = −182.86, *p* < 0.001; Source data are provided as Source Data file (SourceData.xlsx).

Participants (*n* = 25) viewed 10 sequences of 100 images depicting single objects from 10 different semantic categories and performed a manmade/natural decision for each image (Fig. 1a). The sequences of images were pseudorandomly arranged with the constraint that each image was preceded by a different stimulus from either the same or different category, leading to 5 trials per condition per sequence (control condition; Fig. 1a). Behavioral adaptation was evidenced by significantly faster reaction times on targets in the primed vs. control condition overall (Md [IQR]_primed_ = 604 ms [138 ms]; Md [IQR]_control_ = 713 ms [146 ms]; *p* = 2.94^−11^, Wilcoxon signed-rank test). To exclude contributions of mere response priming, we excluded all trials in the control condition that were primed by an image of the different meta-category (manmade/natural). This contrast also revealed evidence in favor of behavioral facilitation (Md [IQR]_primed_ = 605 ms [138 ms]; Md [IQR]_control_ = 682 ms [168 ms]; signed-rank *p* = 6.96^−11^, Wilcoxon signed-rank test). Behavioral adaptation was also evident in response accuracy despite close-to-ceiling performance on the task (Md [IQR]_primed_ = 99.4% [1.1%]; Md [IQR]_control_ = 98.0% [3.0%]; *p* = 1.29^−6^, Wilcoxon signed-rank test). Excluding effects of response priming, in contrast, resulted in no significant effect on response accuracy (Md [IQR]_primed_ = 99.6% [1.1%]; Md [IQR]_control_ = 100.0% [1.21%]; *p* = 0.207, Wilcoxon signed-rank test).

### Reduced neural activity and faster dynamics is evident in iEEG ERPs

Behavioral adaptation was accompanied by NA in grand-average event-related potentials (ERPs) recorded on iEEG in the bilateral amygdalae, hippocampi, and entorhinal and parahippocampal cortices (Fig. 1c). NA evident in grand-averaged ERPs was assessed using cluster-based permutation statistics^46^ and revealed significant decreases in the positive and negative peaks of the ERPs in all anatomical regions we recorded from (amygdala, hippocampus, entorhinal, and parahippocampal cortices). The ERPs for primed versus control targets started to diverge at ~250 ms post-stimulus, which coincides with the onset of neuronal firing in responses to visual stimuli previously reported in single-unit studies of the human MTL^47, 48^.

A prerequisite to test single unit mechanisms of neural adaptation is that prime and target stimuli recruit similar neuronal populations. That this is the case in our data is indicated by higher representational similarity in population responses for primed as compared to control stimuli (Supplementary Fig. 4).

**Fig. 2:**
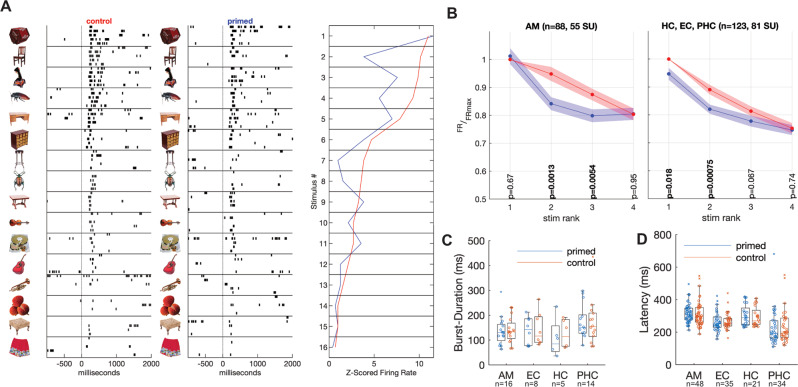
Sharpening in single-unit responses to multiple stimuli. **A** The panel depicts data from one unit. The left two columns depict raster-plots to the 16 stimuli eliciting the strongest responses, separately for the primed and control condition, sorted from top to bottom according to response strength in the control condition. The column on the right depicts the average firing rate during the response-period [0–1 s], normalized to the baseline [−0.5–0 s] as a *Z*-score. Source data are provided as Source Data file (SourceData.xlsx). **B** Averages of all curves in A from the 211 units responding to 4 or more response-eliciting stimuli. A paired *t*-test of primed vs. control tuning curves was performed for each rank on the *x*-axis, and the resulting *p*-values printed in bold if significant at *α* < 0.05. More information on the results of *t*-tests is given in the following. Sample size *n* = 88 units in the amygdala (AM). Rank 1 primed vs. control: *t*(87) = 0.43, CI = [−0.044 0.069], *p* = 0.67; rank 2 primed vs. control: *t*(87) = −3.3, CI = [−0.17 −0.043], *p* = 0.0013; rank 3 primed vs. control: *t*(87) = −2.9, CI = [−0.13 −0.023], *p* = 0.0054; rank 4 primed vs. control: *t*(87)=0.057, CI = [−0.048 0.051], *p* = 0.95. Sample size *n* = 123 units in the hippocampus, entorhinal and parahippocampal cortex (HC, EC, PHC). Rank 1 primed vs. control: *t*(122) = −2.4, CI = [−0.097 −0.0093], *p* = 0.018; rank 2 primed vs. control: *t*(122) = −3.5, CI = [−0.11 −0.03], *p* = 0.00075; rank 3 primed vs. control: *t*(122) = −1.8, CI = [−0.074 0.0026], *p* = 0.067; rank 4 primed vs. control: *t*(122) = −0.33, CI = [−0.045 0.032], *p* = 0.74. Firing rates (FR) for stimuli presented in the primed and control condition in a unit were normalized to the maximum firing rate measured in the control condition (rank 1 stimulus, FRmax). Shaded blue and red areas depict the standard error of the mean. Source data are provided as Source Data file (SourceData.xlsx). **C** Duration of neuronal responses in the primed and control condition. Sample sizes (*n*) indicate the number of units. Center line in boxplots denote the median. Box limits denote the upper and lower quartiles. Whiskers denote the median ± the 1.5 times the interquartile range. Source data are provided as Source Data file (SourceData.xlsx). **D** Response latency was determined using the same Poisson-burst detection algorithm as in **C** for units with a baseline firing rate >2 Hz. For all other units, response onset was determined using the timestamp of the first spike emitted in a time-window ranging from 100 to 1000 ms after stimulus onset. Sample sizes (*n*) indicate the number of units. Center line in boxplots denote the median. Box limits denote the upper and lower quartiles. Whiskers denote the median ± the 1.5 times the interquartile range. Source data are provided as Source Data file (SourceData.xlsx). AM amygdala, HC hippocampus, EC entorhinal cortex, PHC parahippocampal cortex.

To assess evidence of facilitation at the iEEG level, we also assessed differences in peak latencies for the primed and control condition. According to the first two prominent peaks in the grand average ERPs (Fig. 2C), we determined negative peak latency in an early time window (200–400 ms), and positive peaks in a later time window (400–750 ms) for each session and each of the four MTL regions. In the early time window, significantly earlier negative peaks in the primed vs. control condition were evident in the amygdala (Md[iqr]_primed_ = 267[38] ms, Md[iqr]_control_ = 283[31] ms, *p* < 0.001, Wilcoxon signed-rank test), entorhinal cortex (Md[iqr]_primed_ = 263[31] ms, Md[iqr]_control_ = 267[36], *p* < 0.001), and parahippocampal cortex (Md[iqr]_primed_ = 237[45] ms, Md[iqr]_control_ = 253[84], *p* < 0.001). In the later time window, no significant reductions in peak latency for the primed vs. control condition were found (see Supplementary Table 1 for a full list of statistical comparisons).

### Sharpening and fatiguing is evident in single unit activity

To assess single-unit mechanisms of NA, we recorded spiking activity from 4917 units in the MTL. At the neuronal level, sharpening is evident as greater attenuation in spiking for non-optimal than optimal stimuli. Fatiguing, in contrast, is characterized by greater attenuation for optimal than non-optimal stimuli. To decide between the two mechanisms, we selected units responding to multiple stimuli (4 or more; *n* = 211, 136 single units, 75 multi units, see Supplementary Fig. 1a/b for analyses for 2/3 or more responsive stimuli, respectively. See Supplementary Fig. 7 for an analyses using non-normalized firing rates in Hz as dependent measure). For each unit, stimuli were ordered according to the firing rate they elicited during the response period (0–1 s, collapsed over primed and control condition). Curves were normalized per unit to the firing rate during presentation of the most response-eliciting stimulus in the control condition. Primed and control condition were contrasted with signed-rank tests at each of the first four ranks (Fig. 2b). The ranks correspond to the above ordering of stimuli according to the average strength with which made the neuron fire.

In all regions we recorded from, units fired with variable strength to different stimuli, consistent with the idea of a “semantic tuning curve”^28^ rather than an “all-or-nothing” coding of stimulus identity^45^.

In amygdala units, we found a pattern consistent with a sharpening of the tuning in the primed versus control condition (Fig. 2b, left panel). In particular, no significant difference in firing rates between primed and control condition was found for the most response-eliciting stimuli (*p* = 0.67). Attenuation of spiking for primed vs. control condition was evident at response-eliciting stimuli on ranks 2 and 3, and attenuation was stronger in rank 2 when compared to rank 1 (all *p* < 0.01, Fig. 2b).

In other regions of the MTL we recorded from, the pattern was consistent with fatiguing (Fig. 2b, right panel). Here, pairwise comparisons between primed and control condition were significant at ranks 1 and 2, while no pairwise comparison of attenuations between ranks reached significance.

To formally assess that response profiles were significantly different between anatomical regions, we used a 2 × 4 × 2 mixed ANOVA with neurons as units of observation, the within factors Condition (primed, control), Stimulus Rank (1, 2, 3, 4), and Anatomical Region (AM, other MTL regions), and *Z*-scored firing rates as dependent measure. Since we ranked stimuli based on their firing rate, we found significant contrasts for Rank by design. The largest effect was found for the linear contrast of Rank (*F* = 450.426, *p* < 0.001, *η*_p_^2^ = 0.683). As expected, we also found a significant main effect of Condition (*F* = 24.107, *p* < 0.001, *η*_p_^2^ = 0.103). Furthermore, we found significant quadratic interaction contrasts for Rank × Condition (*F* = 12.684, *p* < 0.001, *η*_p_^2^ = 0.057), and Rank × Condition × Region (*F* = 4.864, *p* = 0.029, *η*_p_^2^ = 0.023; see Supplementary Table 2 for a complete list of within-subject contrasts). The significant three-way interaction supports the conclusion that response profiles differ significantly between AM and other regions of the MTL.

To assess the relationship between single unit evidence for sharpening and fatiguing behavior, we correlated firing rate differences between primed and control stimuli on ranks 2 and 3 with behavioral reduction in reaction latencies to primed versus control stimuli (Supplementary Fig. 2). These correlations were insignificant. Furthermore, correlations of the reduction in iEEG signals to primed versus control condition stimuli were uncorrelated with behavioral reaction latency differences (Supplementary Fig. 3). We also correlated reduction of iEEG in with the differences in single unit firing rates between primed and control stimuli on rank 1 (as a measure of fatiguing) and rank 2 (as a measure of sharpening). No significant correlations were observed in these analyses either (Supplementary Fig. 6).

No evidence for facilitation, i.e., the shortening of firing-duration in primed vs. control condition was seen in any of the anatomical regions. Here, we analyzed spike trains in units with a baseline-firing above 2 Hz using a burst detection algorithm to determine firing onset and offset. Comparing the duration of firing between primed and control condition did not reveal significant differences (Fig. 2c). Remarkably, neuronal response onset latencies also did not reflect the behavioral differences in reaction time (Fig. 2d), indicating that these might be caused by facilitation elsewhere in the brain.

To assess whether there is support for the spreading activation model in our data, we compared pre-stimulus activity of units responding to at least one of the stimuli (301 units in the amygdala, and 484 in other MTL regions). The spreading activation model makes no explicit statement about underlying biological mechanisms. One possible prediction might be higher pre-stimulus activity prior to the presentation of the response-eliciting stimulus under the primed condition as compared to the control condition. A paired *t*-test of *Z*-scored firing rates in the time window ranging from −500 ms to stimulus onset (0 ms) in the amygdala was not significant; M(SD)_primed_ = 0.094(0.48), M(SD)_control_ = 0.061(0.45); *t*_300_ = 0.846, *p* = 0.398. The analog analysis for units in other MTL regions did also not show significant effects; M(SD)_primed_ = 0.064(0.47), M(SD)control = 0.027(0.45); *t*_484_ = 1.22, *p* = 0.223 (Supplementary Fig. 5, Supplementary Fig. 8 for the same analyses with raw firing rates as dependent measure). Spreading activation might also manifest in a higher intracranial EEG potential pre-stimulus for the primed vs. control condition. No such effect could be observed in any of the regions we recorded from (Fig. 1C).

## Discussion

Adaptation to information repeating on a highly abstract, semantic level is a feat that is arguably exclusive to human cognition. Three hypothetical mechanisms enabling neural adaptation observed in bulk tissue studies have been proposed at the neuronal level, namely, sharpening, fatiguing, and facilitation. We conducted a semantic priming paradigm with neurosurgical patients implanted with electrodes for the simultaneous recording of iEEG and single-unit activity. We replicated behavioral adaptation at the level of reaction times and NA in bulk-tissue iEEG recordings. Furthermore, evidence suggests that iEEG ERPs are also compressed in time as consequence of adaptation. We did not find evidence for spreading activation as a preparatory mechanism in neither iEEG nor in single unit activity. Patterns of single-unit activity were consistent with the sharpening model in the amygdala and the fatiguing model in other MTL regions. No evidence for the facilitation model was observed on the level of single units.

Our results hence demonstrate that both fatiguing and sharpening elicits NA observed in coarse measures of neural activity. It is conceivable that observed fatiguing in a downstream region is the consequence of sharpening in an upstream region, or that mechanisms of NA could differ between anatomical regions. Furthermore, the cognitive task and the kind of information that is repeated to elicit NA are additional factors that could determine whether neurons in a given brain region display sharpening or fatiguing. Sharpening can be seen a mechanism of reducing noise in neural representations and could be the consequence of top-down gating of relevant information^16^. Sharpening has also been observed in nonhuman-primate studies as a correlate of long-term memory. Sharpening of single neuron IT responses, e.g., have been reported when long-term explicit learning or increased familiarity to visual stimuli is involved^17,18^. Sharpening has also been observed following several days of practice in a delayed match to sample task using degraded visual stimuli in the prefrontal cortex of monkeys^49^. Our results add that sharpening can also be observed for short-term repetition of information at an abstract, semantic level in the human amygdala. We can only speculate as to why the amygdala appears to implement a different single-neuron mechanism of NA than other regions we recorded from, which show patterns consistent with fatiguing. Part of the reason might be that the amygdala is receiving inputs from these other regions showing fatiguing-like patterns of activity. Fatiguing in combination with a gating mechanism upstream could result in non-linear attenuation of activity in response to non-optimal stimuli in the primed as compared to the control condition further downstream in the amygdala.

While it may seem surprising that the amygdala exhibits NA in a conceptual relatedness-priming paradigm, the human single unit literature has long described neurons in the amygdala responding to broad and abstract semantic categories such as images of animals, faces or cars^42^. Likewise, most studies demonstrating units in the human MTL that respond selectively to the semantic content of a stimulus irrespective of presentation modality (e.g., the picture as well as the written and spoken name of an object or a person) found these types of neurons as frequently in the amygdala as in other MTL regions^43,50,51^. This might be due to the fact that these so-called “concept cells”^27^ have mostly been viewed in the light of their potential functional role in declarative memory formation where the anatomical focus was more on the hippocampus and entorhinal cortex, rather than the amygdala. Investigations of the role of concept cells in the context of functions traditionally associated with the amygdala are scarce^35,52^ and may be complicated by the fact that the amygdala in itself is highly heterogenous structure associated with a wide range of functions mostly related to emotions^53^. Thus, we can only speculate that the amygdala benefits from using an abstract semantic code for efficient assessment of external stimuli with regard to their emotional relevance.

Fatiguing, on the other hand, has been observed in primate IT in paradigms entailing short-term repetitions of identical stimuli^21,22^, or back-to-back presentations of similar vs. dissimilar shapes^20^. One previous human study investigating effects of repetition on single unit activity reported a reduction of neural activity from the first to following presentations of the identical response-eliciting stimuli that were separated by several minutes^38^. This study, however, did not distinguish between more and less response-eliciting stimuli and hence could not rule out the possibility that patterns consistent with sharpening were also present. Our results hence critically extend this previous work by showing that fatiguing also results when repetition concerns categorical, semantic information in back-to-back presentations.

Our result of earlier negative peaks in iEEG potentials for the primed vs. control condition are in line with previous studies that found similar evidence for facilitation in intracranial EEG^24,25^. This finding stands in contrast to the lack of evidence for shorter or earlier bouts of activity for primed versus control stimuli in single-unit activity in our data. It is important to keep in mind that especially lower-frequency components of the iEEG signal can also reflect computations happening upstream and should therefore rather be regarded as the input signal into a region. Single-unit activity, on the other hand, is the direct consequence of local computations. It is therefore conceivable that facilitation at this mesoscopic iEEG scale is the result of sharpening and/or fatiguing at the level of microscopic single-unit activity, either in the same region or in upstream regions. In the case of sharpening, this could mean that a smaller population of neurons representing a stimulus more efficiently could result in faster propagation of signals to downstream regions^19^. Sharpening at the single-unit level could thus be regarded as a factor contributing to facilitation such that local networks dynamics settle more quickly into an attractor state^54^. These faster local dynamics could then become observable only on coarser measures such as iEEG, scalp EEG or fMRI. Especially in fMRI, however, faster dynamics (facilitation) are difficult to distinguish from reduced overall activity (fatiguing) or sharpening of representations due to the large temporal integration window of the BOLD signal.

It is noteworthy that the neuronal models for NA (sharpening, facilitation, and fatiguing) were initially formulated in the context of repetition of identical stimuli. In this context, the fatiguing model can only produce fatiguing of neural responses, but it can produce either fatiguing- or sharpening-like effects in (semantic) relatedness priming. If there is little to modest overlap in the neural representations of two related stimuli, then short-term adaptation can reduce the magnitude of the intermediate responses without affecting the peak response much. The more overlap between the first and second stimuli, the more “fatigue-like” the effects can be observed. Our current data set is too limited in the number of trials eliciting neuronal responses to be further subdivided in the ones with high vs. low overlap other than already inherent in our experimental manipulation. Future studies could parametrically vary the overlap of neuronal populations recruited by prime and target stimuli to further investigate this question.

NA was observed in intracranial EEG while underlying single-unit mechanisms could investigated at the same time. Previous studies of NA in the human MTL were performed with fMRI, and revealed NA in the context of higher forms of cognition such emotion recognition^55^, episodic memory^39^, and semantic processing^41^. There are studies assessing models of sharpening, facilitation and fatiguing with fMRI, reporting evidence for facilitation and sharpening, depending on cortical region and task^8,56^. These studies investigated NA in the context of face and object perception, and phenomena modeled in these fMRI studies are confined to cortical regions exhibiting macroscopic topographic organization such as neocortical regions involved in the storage of semantic knowledge^57^. Macroscopic topographic organization, however, is a feature that is not present in the human MTL such as e.g. the hippocampus or the amygdala^58,59^. Our results hence extend these findings with evidence for fatiguing and sharpening in regions of higher-order cognition that are lacking macroscopic topographical organization.

Single-unit studies in nonhuman primates, in contrast, mainly investigated lower-level perceptual processes and recorded single-unit activity only^17,18,21,22^. Connecting findings from non-human single-unit studies with human fMRI studies has been difficult as different species, tasks and anatomical regions were investigated. Having data on both the micro- and the meso-scale simultaneously hence critically extends previous work as we report NA on the meso-scale together with data on the micro-scale in the same anatomical regions of the same subjects performing the same task.

The dominating psychological model for semantic-relatedness priming is the spreading activation model^10^. In our data we do not find evidence in favor of this model as neither iEEG nor single-unit activity was increased pre-stimulus in the primed vs. the control condition. As the spreading activation model leaves the biological implementation open, it is conceivable that the measures we record are insensitive to residual activity spreading through the network. For example, spreading activation could result in subthreshold activity, i.e., excitatory or inhibitory postsynaptic potentials (E/IPSPs). More recent computational models explain NA and behavioral facilitation in terms of, e.g., predictive coding^4^ and Bayesian surprise^60^. While most of these models of high-level abstract cognition such as sentence understanding usually remain vague with respect to their precise biological implementation, the latter sentence gestalt model^60^ suggests that as semantic knowledge and expectations are updated by repeated exposures of semantic relationships to the network, a shift of labor from unit activity to synaptic connections may be responsible for overall reduced spiking activity for less surprising (primed) stimuli. Both the measurement of EPSPs and IPSPS for the spreading activation model, as well as measurements of synaptic strength unfortunately still go beyond what is currently possible to assess in human intracranial in-vivo electrophysiology.

Finally, our results speak to the debate whether single units in the human MTL fire in a binary, all-or-none fashion to semantic concepts^45^, or whether these units display graded responses to multiple stimuli, resembling classical tuning curves observed in perceptual domains^61^. Consistent with the latter idea of tuning curves along semantic dimensions^28^, we found units responding with graded intensities to different stimuli (see Fig. 2A), also of the same semantic category^28^. Previous studies interpreting responses as binary to one or more concepts, thus corresponding to a boxcar-shaped tuning curve, assessed tuning only for heterogenous sets of visual stimuli optimized for identifying as many units as possible responding to one of the stimuli in the set^43,45,62^. The stimulus set in this and a previous study^28^ entailed images that can systematically be grouped into rather narrow semantic categories such as “insects” or “clothes”, making detection of rather narrow semantic tuning to different members of a semantic category more feasible.

## Methods

### Participants

Participants were 25 neurosurgical patients (9 female; 19–62 years of age, *M* = 38, *SD* = 13) implanted with depth electrodes for chronic seizure monitoring to identify seizure onset zones for later surgical removal. The length of stay on the epilepsy monitoring unit was ~7–10 days during which multiple cognitive experiments were conducted. The study was approved by the Medical Institutional Review Board of the University Bonn. Each patient gave informed written consent. No compensation for participation was paid.

### Statistical tests

All statistical tests are two-tailed. Unless stated otherwise, statistical tests rendering an *α* value below 0.05 are considered significant.

### Task and stimuli

Stimulus material consisted of 100 images from 5 manmade and 5 natural categories of 10 exemplars each. One session was subdivided into ten runs. In each of the ten runs, all 100 images were presented once as target, and one image was presented once additionally in the beginning of the run to serve as prime for the first stimulus in the sequence of 100. This very first presentation was discarded from analyses, resulting in 10 presentations of each of the 100 images as target in total. The sequences were pseudorandomly arranged such that in 5 presentations, the image was preceded by an image of the same category (primed condition) and 5 times preceded by an image of a different category.

A trial started with the presentation of a blank screen for 200–400 ms (random jitter), followed by a fixation dot (300 ms). Then the image was displayed on screen until the subject pressed either the left or right arrow button to indicate whether the depicted object is something manmade or natural, respectively.

### Analysis of behavioral priming

Reaction times of individual trials shorter than the mean reaction times of a session (M) −2.5 times the standard deviation (SD), or longer than the M + 2.5 SD were excluded from further analyses. We then averaged reaction times across trials of each condition/participant combination and performed a signed-rank test for the contrast primed vs. control condition, treating values from the same participant as pairs.

### Electrophysiological recordings

Data was recorded from Behnke–Fried depth electrodes (AdTech, Racine, WI) equipped with eight macro-contacts (iEEG), and a bundle of nine microwires for single unit recordings that were inserted in the shaft of the implant and protruded ~4 mm from its tip into the targeted brain region. A bundle of microwires consisted of eight high-impedance recording electrodes and one low-impedance reference. Signals from micro and macro contacts were amplified and recorded by a Neuralynx ATLAS system (Bozeman, MT). The sampling rate was 2 kHz for the macro and 32 kHz for the micro channels. All microwire signals were referenced against one or multiple of the low-impedance reference microwires, depending on signal quality. One macro-contact was chosen as common reference for all macro-recording channels based on clinical considerations. Macro recordings were then re-referenced offline to linked mastoid electrodes.

### iEEG analysis

Data recorded from the most medial contact was chosen for iEEG analysis as this contact lies closest to microwire recordings. After offline re-referencing to linked-mastoids, data from macro contacts were downsampled to 256 Hz and then bandpass-filtered between 0.1 and 80 Hz. Data was segmented from −1 to 2 seconds relative to stimulus onset, and baseline corrected by subtracting the mean signal from −200 to 0 in a segment from every data point in that segment. To exclude segments containing non-neural artefacts, we computed for each segment the ratio of the maximum of the absolute signal in each segment to the median of all absolute maxima across segments. Segments in which this ratio exceeded 2.5 were excluded from further analyses. Similarly, to estimate the noise floor in an individual segment of data, we calculated the median of the absolute values of all samples in a segment. We then took the median of the distribution of these medians across all segments. Finally, we divided individual medians by the median of all medians. Segments in which this ratio exceeded 2.5 were excluded from further analyses. Event-related potentials were then computed by averaging segments per experimental condition (primed vs. control), and by averaging across recording sites.

### Cluster-based permutation statistics

To test for significant differences in grand-average ERPs, we used a cluster-based label shuffling procedure introduced by Maris & Oostenveld^46^. Briefly, a t-test was conducted at each sample point between individual traces going into the grand-average of the primed and control condition. The tests were conducted once with correct labels assigned to the iEEG traces, and 1000 times with random assignment of labels to the traces. Clusters were defined as consecutive sample-points at which the t-test exceeded a cluster *α* level of 0.001. To determine whether a cluster reached significance, the sum of t-value *s* in a cluster resulting from correct assignment of the labels to the data had to exceed the 99^th^ percentile or fall below the first percentile of the distribution of sums of t-values resulting from label-shuffled data.

### Spike sorting

The software packages wave_clus^63^ and Combinato^64^ were used to sort action potentials. Wave_clus was used in the first 33 sessions, and Combinato for the following 26 sessions because we switched to using Combinato for reasons unrelated to this work. Spike sorting was manually optimized immediately after recording because the paradigm was also used to screen for response-eliciting stimuli in the morning of a day of testing for ensuing experiments. In total, we recorded from 4917 units of which 2009 were classified as single units (SU, 41%). In all, 1392 units were recorded in the amygdala (656 SU), 1863 units (706 SU) in the hippocampus, 828 (228 SU) in the entorhinal cortex, and 831 units (319 SU) in the parahippocampal cortex.

### Normalization of firing rates

All statistics on unit activity were conducted with normalized firing rates. Raw firing rates of unit activity during the activation period (100–1000 ms post-stimulus) were normalized using a z-score with respect to the mean and the standard deviation of the distribution of firing rates of all 1000 trials collapsed over condition (primed, control) during a pre-stimulus baseline period ranging from −500 to 0 ms.

### Binwise rank-sum criterion

The criterion to identify stimuli eliciting a significant response has previously been used^47^. Spikes were counted in 19 overlapping 100 ms bins ([0:100:1000], and [50:100:950] ms after stimulus onset), and a rank-sum test was performed on each bin between the distribution of spike rates in this bin over the 10 trials (collapsed over primed and control condition) in which the stimulus was shown and the distribution of spike rates during the baseline (−500 to 0 ms) of all 1000 trials. The Simes procedure was used to correct for multiple (19) comparisons. A stimulus was considered as response-eliciting, if (a) one or more of the 19 rank-sum tests were significant at the level of *α* = 0.001, (b) response-period firing (0 to 1000 ms) was higher than baseline (0 to −500 ms), and (c) one or more spikes were fired in the response-period in at least half of the trials in which the stimulus was presented.

### Reporting summary

Further information on research design is available in the Nature Portfolio Reporting Summary linked to this article.

## Supplementary information

Supplementary Information

Reporting Summary

## Data Availability

Relevant data are available here: https://github.com/rebrowski/neuralAdapatationInMTL. Source data presented in the figures are also provided with this paper.

## Source data

Source Data

## Code availability

Custom code to analyze the data is available here: https://github.com/rebrowski/neuralAdapatationInMTL.
