# Supplementary material for: Single-neuron mechanisms of neural adaptation in the human temporal lobe
Source: Nat Commun. 2023 Apr 29;14:2496. doi: 10.1038/s41467-023-38190-5 (PMC10148801; doi:10.1038/s41467-023-38190-5)
Supplement: Supplementary file 1 — Supplementary Information [file 41467_2023_38190_MOESM1_ESM.pdf]

SUPPLEMENTAL INFORMATION

Single-neuron mechanisms of neural adaptation in the human temporal lobe.

Thomas P. Reber[1,2*], Sina Mackay[2], Marcel Bausch[2], Marcel Kehl[2],
Valeri Borger[3], Rainer Surges[2], Florian Mormann[2]

[1] Faculty of Psychology, UniDistance Suisse, Brig, Switzerland
[2] Department of Epileptology, University of Bonn Medical Centre, Bonn, Germany
[3] Department of Neurosurgery, University of Bonn Medical Centre, Bonn, Germany

Author Note

[*] Correspondence: treber@live.com. All authors declare no conflicts of interest.

Key words: Semantic priming, single units, long-term memory, intracranial EEG, repetition suppression

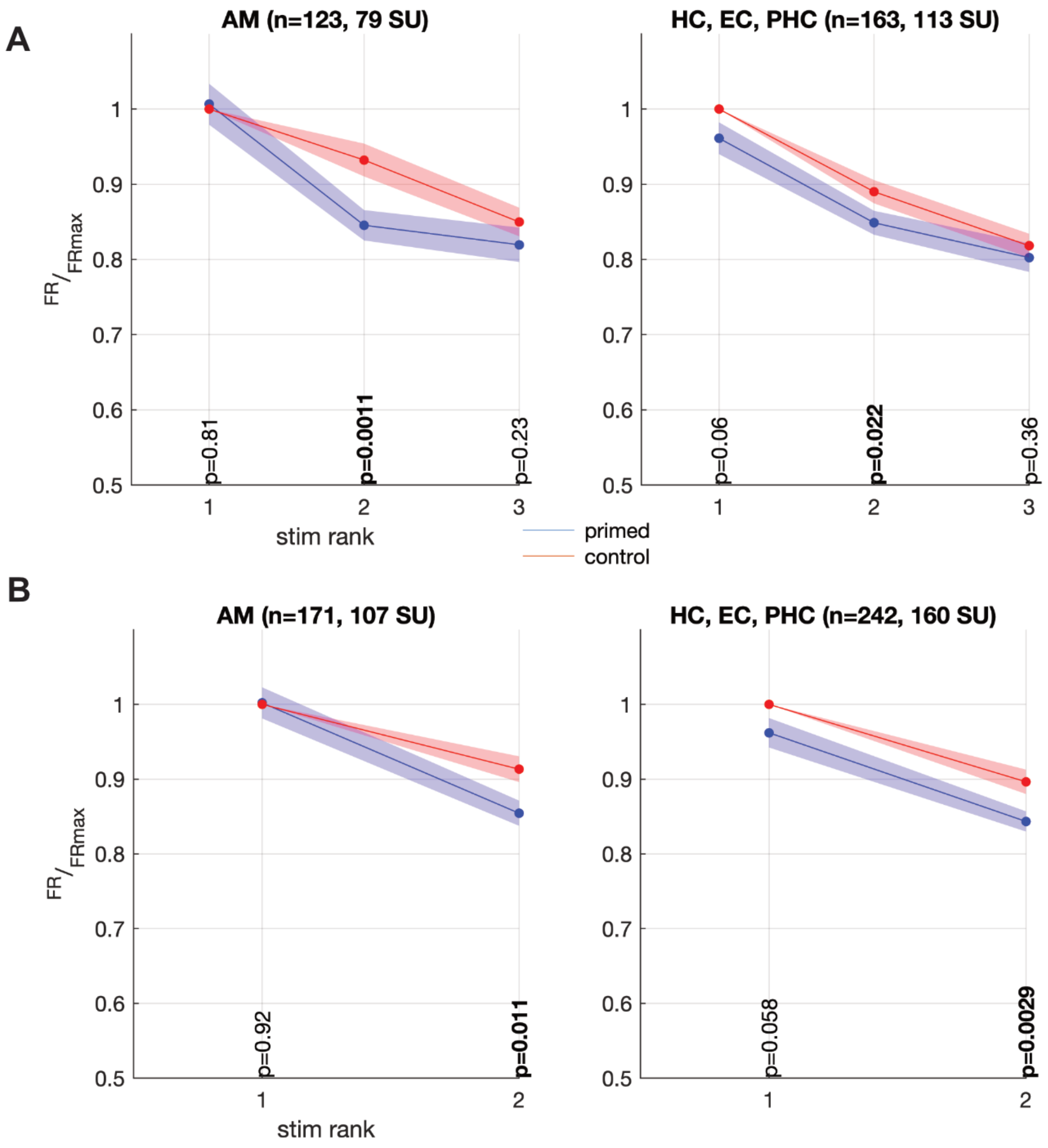

Supplementary Figure 1.

A. Averages of all curves in A from the 286 units responding to 3 or more response-eliciting stimuli. Shaded blue and red areas depict the standard error of the mean. Uncorrected two-tailed paired t-tests of primed vs. control tuning curves was performed for each rank on the x-axis, and the resulting p-values printed in bold if significant at alpha < 0.05. Sample size n = 123 units in the amygdala (AM). Rank 1 primed vs. control: t(122)=0.25, CI=[-0.046 0.059], p = 0.81; rank 2 primed vs. control: t(122)=-3.3, CI=[-0.14 -0.035], p = 0.0011; rank 3 primed vs. control:

t(122)=-1.2, CI=[-0.08 0.02], p = 0.23; Sample size n = 163 in hippocampus (HC), entorhinal cortex (EC), and parahippocampal cortex (PHC). Rank 1 primed vs. control: t(162)=-1.9, CI=[-0.079 0.0017], p = 0.06; rank 2 primed vs. control: t(162)=-2.3, CI=[-0.077 -0.0059], p = 0.022; rank 3 primed vs. control: t(162)=-0.93, CI=[-0.049 0.018], p = 0.36. Source data is available in SourceData.xlsx.

B. Averages of all curves in A from the 413 units responding to 2 or more response-eliciting stimuli. Shaded blue and red areas depict the standard error of the mean. Uncorrected two-tailed paired t-tests of primed vs. control tuning curves was performed for each rank on the x-axis, and the resulting p-values printed in bold if significant at alpha < 0.05. Sample size n = 171 units in the amygdala (AM). Rank 1 primed vs. control: t(170)=0.098, CI=[-0.039 0.043], p = 0.92; rank 2 primed vs. control: t(170)=-2.6, CI=[-0.1 -0.014], p = 0.011. Sample size n = 242 units in the hippocampus (HC), entorhinal cortex (EC), and parahippocampal cortex (PHC). Rank 1 primed vs. control: t(241)=-1.9, CI=[-0.077 0.0014], p = 0.058; rank 2 primed vs. control: t(241)=-3, CI=[-0.088 -0.018], p = 0.0029. Source data is available in SourceData.xlsx.

Region	Time Window (ms)	Polarity	Peak Latency (Md[iqr] ms)		Signed rank p
			Primed	Control	
AM	200-400	neg	267[38]	283[31]	.000939*
AM	400-750	pos	537[188]	557[100]	.0618
HC	200-400	neg	275[111]	290[74]	.0996
HC	400-750	pos	690[322]	705[238]	.142
EC	200-400	neg	263[31]	267[36]	.000168*
EC	400-750	pos	506[226]	557[114]	.193
PHC	200-400	neg	237[45]	253[84]	5.02×10^{-5}*
PHC	400-750	pos	592[209]	586[162]	.0631

Supplementary Table 1: Peak Latencies in iEEG ERPs *: p<.05, AM: Amygdala, HC: Hippocampus, EC: Entorhinal Cortex, PHC: Parahippocampal Cortex

Source	Rank	Condition	F	p	η_p^2
Rank	linear		450.426	4.7381×10^{-54}*	.683
	quadratic		74.086	1.8054×10^{-15}*	.262
	cubic		8.422	.004*	.039
Condition		linear	24.107	0.000002*	.103
Condition × Region		linear	.003	.956	.000
Rank × Region	linear		1.125	.290	.005
	quadratic		.206	.650	.001
	cubic		.609	.436	.003
Rank × Condition	linear	linear	1.361	.245	.006
	quadratic	linear	12.684	0.000457 *	.057
	cubic	linear	1.062	.304	.005
Rank × Condition × Region	linear	linear	1.424	.234	.007
	quadratic	linear	4.864	.029*	.023
	cubic	linear	.096	.757	.000

Supplementary Table 2: 2 × 4 × 2 mixed ANOVA with neurons as units of observation, the within factors Condition (primed, control), Stimulus Rank (1, 2, 3, 4), and Anatomical Region (AM, other MTL regions), and Z-scored firing rates as dependent measure.

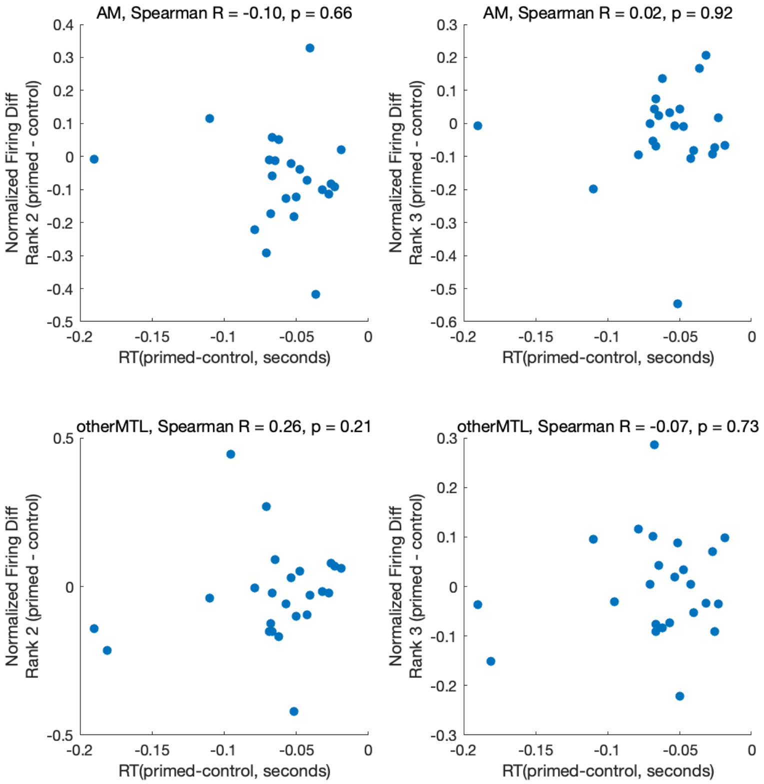

Supplementary Figure 2.

We correlated the mean difference in firing for each pair of rank 2 and 3 stimuli for the primed minus control condition with reaction time differences (primed minus control) for each subject. No significant correlations were found between these two measures in either the amygdala or other regions in the MTL, and neither for rank 2 nor for rank 3 stimuli. Please note that this analysis would only detect rather strong correlations given the unit of observations are participants rather than units. The N is therefore much lower than for analyses across neurons as units of observation. AM: units in the Amygdala, otherMTL: units in the hippocampus, entorhinal and parahippocampal cortex. Source data are provided as Source Data file (SourceData.xlsx).

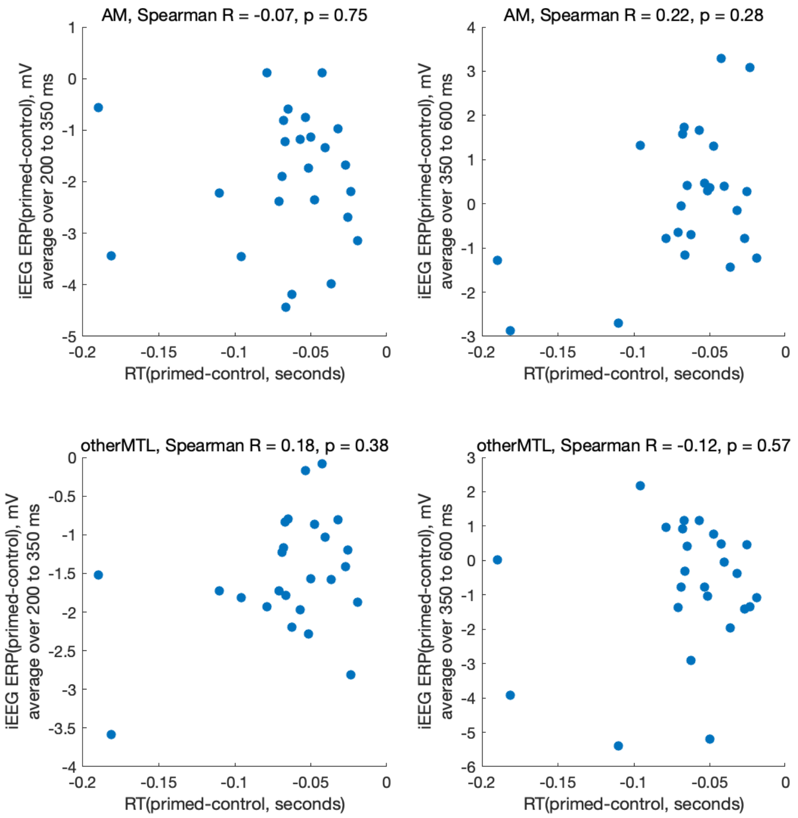

Supplementary Figure 3.

We calculated the difference in iEEG ERPs for primed vs. control stimuli in a time-window from 200-350 ms, and in a time window from 350-600 ms (corresponding to the first two ERP components we observed in the iEEG). Again, no significant correlations were found with reaction time differences of primed minus control condition. Source data are provided as Source Data file (SourceData.xlsx).

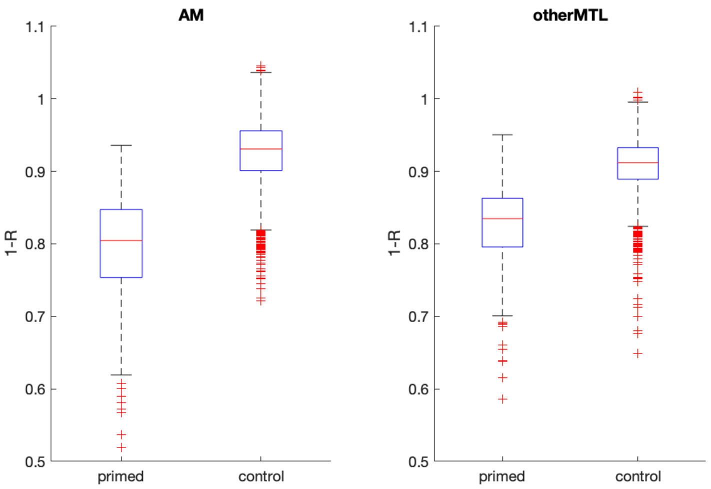

Supplementary Figure 4.

Representational dissimilarity (1-R) for pairs of stimuli grouped by condition. Primed-condition stimulus pairs consist of two images from the same semantic category (e.g., two wild animals). Control condition pairs consist of stimuli from different semantic categories (e.g., a musical instrument and a wild animal). Source data are provided as Source Data file (SourceData.xlsx).

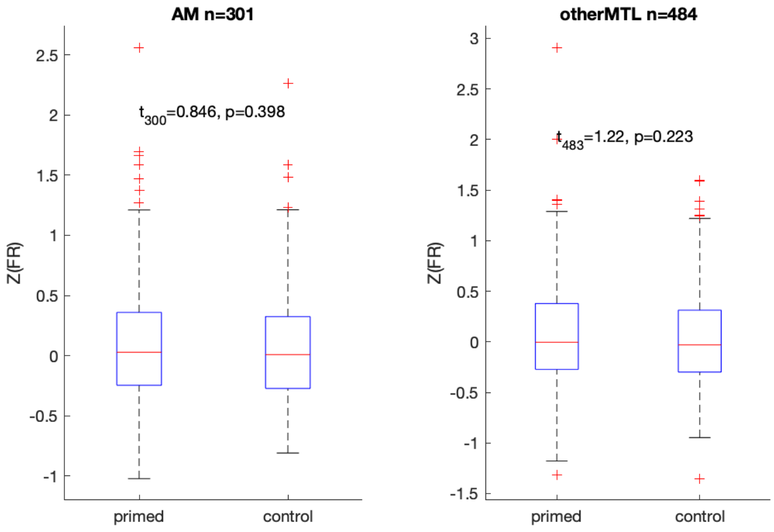

Supplementary Figure 5.

Pre-stimulus (-500 ms to 0 ms) normalized firing of units in the Amygdala (AM) and units in other regions of the MTL we recorded from (entorhinal, parahippocampal cortices and hippocampus). The p- and t-value depicted result from two-tailed pairwise t-tests between the primed and control condition. Source data are provided as Source Data file (SourceData.xlsx).

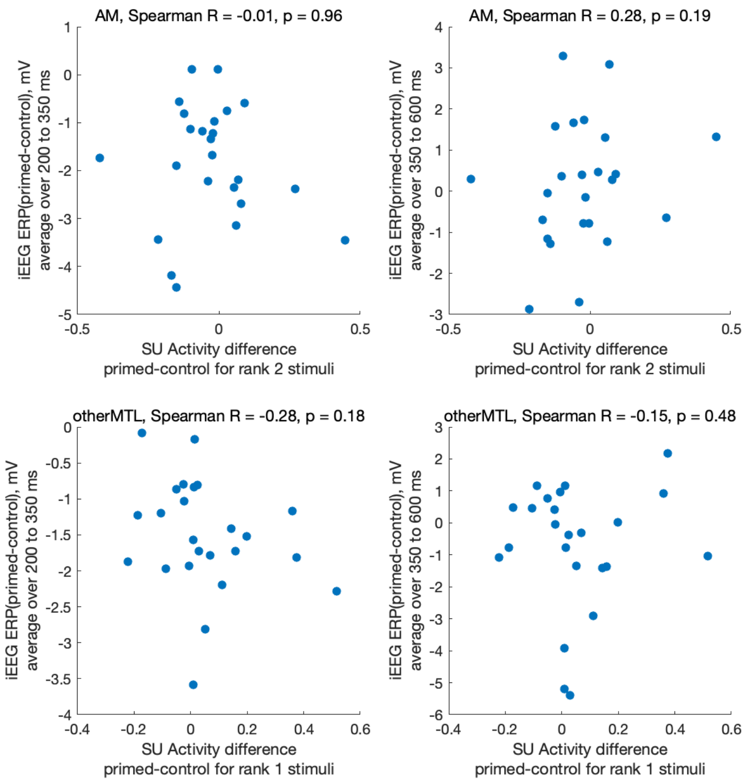

Supplementary Figure 6.

Correlations of ERP differences between primed and control condition with differences in unit firing at rank 2 in the amygdala (as a measure of sharpening) and at rank 1 in other MTL regions (as a measure of fatiguing). Source data are provided as Source Data file (SourceData.xlsx).

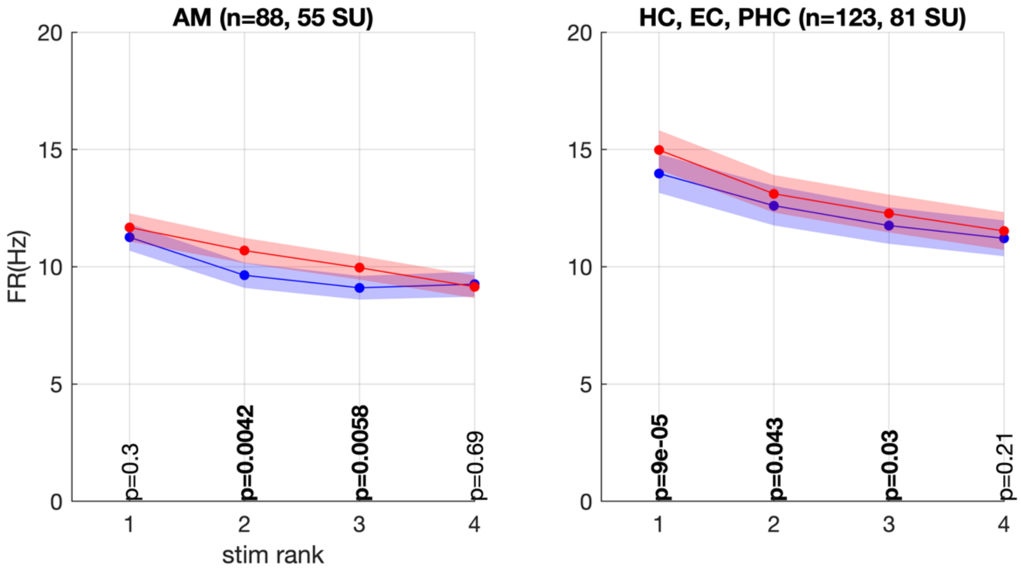

Supplementary Figure 7. Same as Figure 2B but using non-normalized firing rates in Hz as dependent measure. Averages of all curves in A from the 211 units responding to 4 or more response-eliciting stimuli. A paired t-test of primed vs. control tuning curves was performed for each rank on the x-axis, and the resulting p-values printed in bold if significant at alpha < 0.05. Shaded blue and read areas depict the standard error of the mean. Source data are provided as Source Data file (SourceData.xlsx).

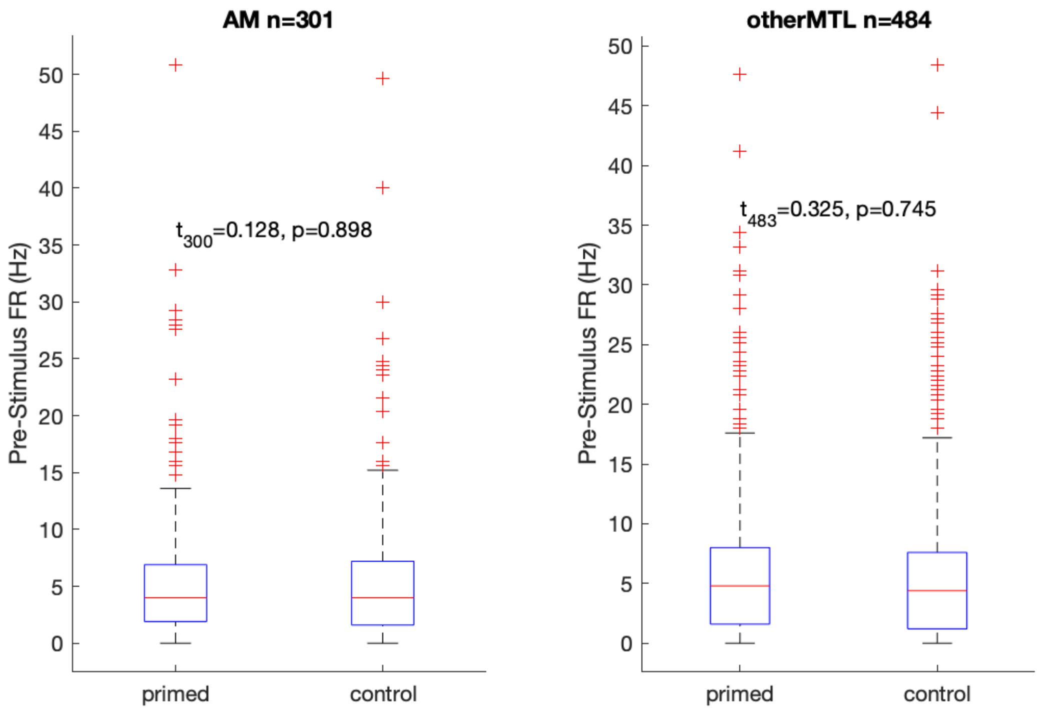

Supplementary Figure 8

Pre-stimulus (-500 ms to 0 ms) non-normalized firing in Hz of units in the Amygdala (AM) and units in other regions of the MTL we recorded from (entorhinal, parahippocampal cortices and hippocampus). The p- and t-value depicted result from two-tailed pairwise t-tests between the primed and control condition. Source data are provided as Source Data file (SourceData.xlsx).
