# Supplementary material for: Single-neuron mechanisms of neural adaptation in the human temporal lobe
Source: Nat Commun. 2023 Apr 29;14:2496. doi: 10.1038/s41467-023-38190-5 (PMC10148801; doi:10.1038/s41467-023-38190-5)
Supplement: Supplementary file 2 — Reporting Summary [file 41467_2023_38190_MOESM2_ESM.pdf]

nature portfolio

Corresponding author(s): Thomas P. Reber

Last updated by author(s): 3.3.2023

Reporting Summary

Nature Portfolio wishes to improve the reproducibility of the work that we publish. This form provides structure for consistency and transparency in reporting. For further information on Nature Portfolio policies, see our Editorial Policies and the Editorial Policy Checklist.

Statistics

For all statistical analyses, confirm that the following items are present in the figure legend, table legend, main text, or Methods section.

n/a	Confirmed	
☐	☒	The exact sample size (*n*) for each experimental group/condition, given as a discrete number and unit of measurement
☐	☒	A statement on whether measurements were taken from distinct samples or whether the same sample was measured repeatedly
☐	☒	The statistical test(s) used AND whether they are one- or two-sided<br>*Only common tests should be described solely by name; describe more complex techniques in the Methods section.*
☒	☐	A description of all covariates tested
☒	☐	A description of any assumptions or corrections, such as tests of normality and adjustment for multiple comparisons
☐	☒	A full description of the statistical parameters including central tendency (e.g. means) or other basic estimates (e.g. regression coefficient) AND variation (e.g. standard deviation) or associated estimates of uncertainty (e.g. confidence intervals)
☐	☒	For null hypothesis testing, the test statistic (e.g. *F*, *t*, *r*) with confidence intervals, effect sizes, degrees of freedom and *P* value noted<br>*Give P values as exact values whenever suitable.*
☒	☐	For Bayesian analysis, information on the choice of priors and Markov chain Monte Carlo settings
☒	☐	For hierarchical and complex designs, identification of the appropriate level for tests and full reporting of outcomes
☒	☐	Estimates of effect sizes (e.g. Cohen's *d*, Pearson's *r*), indicating how they were calculated

Our web collection on statistics for biologists contains articles on many of the points above.

Software and code

Policy information about availability of computer code

Data collection	We used a Neuralynx ATLAS system running Cheetah and Pegasus Software to record Electrophysiological signals. We used psychtoolbox 3 running on a debian operating system to present visual stimuli.
Data analysis	The software packages wave_clus (Quian Quiroga et al., 2004) and Combinato (Niediek et al., 2016) were used to sort action potentials. All further analyses were performed using MATLAB and custom scripts that are available on https://github.com/rebrowski/neuralAdapatationInMTL .

For manuscripts utilizing custom algorithms or software that are central to the research but not yet described in published literature, software must be made available to editors and reviewers. We strongly encourage code deposition in a community repository (e.g. GitHub). See the Nature Portfolio guidelines for submitting code & software for further information.

Data

Policy information about availability of data

All manuscripts must include a data availability statement. This statement should provide the following information, where applicable:

- Accession codes, unique identifiers, or web links for publicly available datasets
- A description of any restrictions on data availability
- For clinical datasets or third party data, please ensure that the statement adheres to our policy

Data and code is made available and an availability statement is included in the revised manuscript.

Human research participants

Policy information about studies involving human research participants and Sex and Gender in Research.

Reporting on sex and gender	Participants were 25 neurosurgical patients (9 female; 19 to 62 years of age, M = 38, SD = 13).
Population characteristics	Participants were epilepsy patients implanted with depth electrodes for chronic seizure monitoring.
Recruitment	Participants were asked whether they would be willing to participate in our study after the decision has been made to implant electrodes for medical reasons.
Ethics oversight	The study was approved by the Medical Institutional Review Board of the University Bonn.

Note that full information on the approval of the study protocol must also be provided in the manuscript.

Field-specific reporting

Please select the one below that is the best fit for your research. If you are not sure, read the appropriate sections before making your selection.

☐ Life sciences ☒ Behavioural & social sciences ☐ Ecological, evolutionary & environmental sciences

For a reference copy of the document with all sections, see nature.com/documents/nr-reporting-summary-flat.pdf

Behavioural & social sciences study design

All studies must disclose on these points even when the disclosure is negative.

Study description	This is a quantitative study (electrophysiological recordings during behavioural experiments). We investigate the single neuron and iEEG correlates of semantic priming and neural adaptation.
Research sample	Participants were 25 neurosurgical patients (9 female; 19 to 62 years of age, M = 38, SD = 13) implanted with depth electrodes for chronic seizure monitoring to identify seizure onset zones for later surgical removal.
Sampling strategy	We obtained a convenience sample of Patients admitted to the hospital between 2015 and 2017. The sample size of 4917 units from 25 patients exceeds typical sample sizes involving epilepsy patients in comparable studies.
Data collection	We used a Neuralynx amplifier to record from intracranial electrodes of epilepsy patients that were seated in a hospital bed while operating a laptop computer displaying visual stimuli to which the patients reponded by button press, which were recroded to the laptop computer.
Timing	Data collection started May 2014 until February 2017.
Data exclusions	no data was excluded
Non-participation	no dropouts
Randomization	Between subject randomization was not used in this study.

Reporting for specific materials, systems and methods

We require information from authors about some types of materials, experimental systems and methods used in many studies. Here, indicate whether each material, system or method listed is relevant to your study. If you are not sure if a list item applies to your research, read the appropriate section before selecting a response.

Materials & experimental systems

n/a	Involved in the study
☒	☐ Antibodies
☒	☐ Eukaryotic cell lines
☒	☐ Palaeontology and archaeology
☒	☐ Animals and other organisms
☒	☐ Clinical data
☒	☐ Dual use research of concern

Methods

n/a	Involved in the study
☒	☐ ChIP-seq
☒	☐ Flow cytometry
☒	☐ MRI-based neuroimaging
